# Understanding how to improve physicians’ paradigms for prescribing antibiotics by using a conceptual design framework: a qualitative study

**DOI:** 10.1186/s12913-018-3657-x

**Published:** 2018-11-14

**Authors:** Egui Zhu, Uno Fors, Åsa Smedberg

**Affiliations:** 1 Department of Learning, Informatics, Management and Ethics, Karolinska Institutet, Stockholm, Sweden; 2 Faculty of Education, Hubei University, Wuhan, China; 3 Department of Computer and Systems Sciences, Stockholm University, Stockholm, Sweden

**Keywords:** Antimicrobial resistance, Primary care, Augmented reality, Continuing professional development

## Abstract

**Background:**

Antimicrobial resistance (AMR) is a growing public health threat. Primary care physicians are important inducers of the overuse of antimicrobials and inappropriate prescribing. Augmented reality (AR) might provide a potential educational tool in health care. The aim of this study was to identify the need for education and expectations for AR-based education in the context of improving the rational use of antibiotics by primary care physicians in China.

**Methods:**

The study used a qualitative approach based on face-to-face interviews with eleven physicians from three community health service centers and stations in China. We used a hybrid thematic analysis approach to analyze the interview data. A conceptual design framework, mobile augmented reality education (MARE), guided the work.

**Results:**

The physicians’ personal prescription paradigms included problems regarding the way they diagnosed and chose treatments and prescriptions. Although the physicians mentioned that they should not treat patients with antibiotics without proof of a bacterial infection, in practice, they often did not wait for necessary test results before they prescribed antibiotics. It was also revealed that they often experienced difficulties when trying to convince patients to follow non-antibiotic treatments. Physicians’ prescription of antibiotics was based on three different paths: if they thought there was a bacterial infection, if they thought preventing additional possible infections for the patient to be necessary; and if the patients requested antibiotics. The physicians expressed various learning needs for the rational use of antibiotics, and their expectations of an AR-based educational intervention included suggestions for contents, learning assets, learning environments and learning activities.

**Conclusions:**

The results showed that the physicians were not only unfamiliar with national guidelines on the use of antibiotics and local AMR patterns but also had personal paradigm issues related to the physicians’ decision making. Moreover, the physicians provided meaningful insights into and expectations for possible AR-based education on AMR. In this article, we demonstrate how to apply the MARE framework to analyze the needs of educational interventions for rational use of antibiotics.

## Background

Antimicrobial resistance (AMR) is a growing public health threat [1, 2]. Multiple studies have shown that the use of antibiotics in primary care settings is an important contributing factor behind AMR [3, 4]. It has been pointed out that there is a need for more effective models for continuing professional development (CPD) in order to address behavior modification and not just to provide correct knowledge [5]. Transformative learning theory suggests that adult learners’ actions are determined by their frame of reference which has been formed over previous experience [6]. Thus, effective CPD could combine the interventions with a suitable learning environment for improving GPs’ decision making and addressing problems of their frame of reference. The World Health Organization (WHO) has published a guide for good prescribing in which the decision making of prescribing is defined as P-diagnosis, P-treatment, P-prescription and P-drug, where P stands for personal [7]. The WHO guide could be used to identify this type of problems.

A variety of technologies are available to enhance CPD. Augmented reality (AR) is defined as computer-generated contents, such as sound, text, animation, 3D or graphics, etc., to augment a live view of a real-world environment shown through the same interface [8]. In educational settings, AR can be used in different ways to support learning [9]. AR adds specific information about the location for students to learn about the places they visit, to play AR games in simulated situations and to solve problems together [10]. AR applications have been used to increase students’ motivation to learn [11] and people’s motivation to walk outdoor which has significant implications for public health [12]. AR is also used to provide real-life objects with virtual information for instructions during maintenance and repair work [13]. In our integrative review, 96% of 25 included papers showed that AR might be beneficial for healthcare professional educational development in varying medical subjects [14]. In addition, our previous study of primary care physicians in China showed that the physicians had a positive attitude to AR which they viewed as potentially useful in their CPD, and relational use of medicine was one topic of their suggestion [15]. However, we did not find that AR has been used in CPD for GPs.

As the overuse and misuse of antibiotics and other drugs is a well-known problem in Chinese primary care [16, 17], we saw a need to explore the potential use of AR to address this problem. Many previously published studies have focused on analyzing the factors of misuse or overuse of antibiotics [18]; few have explored how physicians make decisions and how to improve GPs’ competence and practices. To guide the design and development of AR for medical education, we have designed a conceptual framework, the mobile augmented reality education (MARE) [19]. In the MARE framework, the design of AR-based CPD is based on learner-centered design that requires understanding of learners’ personal paradigms as well as their expectations of AR. The paradigm concept was first used by Kuhn for scientific inquiry but was adapted as a frame of reference by Mezirow in transformative learning theory [20]. We use the concept of ‘personal paradigm’ to refer to the personal style of decision making which WHO defined as the concepts P-diagnosis, P-treatment, P-prescription and P-drug [7]. Therefore, the MARE framework might be used to guide us to discover the AR-based CPD design needs for Chinese primary care physicians.

### Objectives

This study aimed to identify the CPD needs in the context of rational use of antibiotics by primary care physicians in China. The following research questions were addressed:

- RQ1: What are the current personal paradigms for prescribing antibiotics among primary care physicians in China, and how might these paradigms be improved by using e-learning and AR-based CPD?
- RQ2: What are physicians’ expectations for AR-based CPD related to prescribing antibiotics?

## Methods

### Context

Primary care in China is provided through community health service centers or stations (CHCSs) in urban areas but through township hospitals or village clinics in rural areas [21]. In Wuhan, a large city with more than 10 million people in the center of China, there is a total of 540 CHCSs [22]. This study was conducted at three CHCSs in Wuhan. Wuhan is the capital of the Hubei province, which reported the highest level of irrational use of injection antibiotics among the six provinces in China [16]. It was one of ten pilot cities for the introduction of the Chinese national GP service mode reform in 2012 [23].

### Study design

A qualitative approach [24], based on interviews with physicians at the random selected CHCSs, was used to investigate physicians’ perceptions regarding prescribing and learning, their decision processes and their expectations of using AR within real-work contexts. We wanted to obtain a holistic and deep understanding regarding what the physicians wanted to learn based on their experience and personal learning preferences, and what they needed to learn based on their personal paradigms. The purpose of the interviews was also to identify important aspects of AR design needs for primary care physicians, aiming to improve their rational use of antibiotics (RQ1 and RQ2). The interviews were conducted during the physicians’ routine daytime work hours by the first author (EZ) so she could observe in connection with the interviews how the physicians made decisions about prescribing antibiotics. We considered the WHO guide as a standard of good prescription practice and we used it only as a vehicle to identify the personal paradigms of the interviewees. In order to be able to answer the research questions, only physicians working in CHCSs and allowed to prescribe antibiotics were included. The sampling strategies suggested by Robinson for interview-based qualitative research were used [25].

### Data collection

Data was collected from December 2014 to January 2015 through individual face-to-face interviews by EZ. Eleven physicians (of whom three were male and eight females) from three CHCSs were recruited. Among the eleven participants, the first participant was recruited through convenience sampling and the rest were recruited by snowball sampling, until saturation was reached. The first part of the interview focused on understanding the possible personal prescription paradigm problems. We identified the paradigm problems of their prescription styles that did not follow the WHO guide standard (and all its sub-standards). The second part was used to allow the physicians to express their expectations for AR-based CPD to improve the prescription paradigm. As no AR application for drug prescription training was available at the time of the interviews, another type of AR app for medical training, the free APP PlayAR Human Anatomy 4D for iPad (version 1.4.04, PlayAR Games, United States), was shown to the physicians during the last part of the interview to exemplify medical AR applications for learning when they formulated their expectations for a possible AR-based CPD for AMR. The AR system example allowed the physicians to look at various organ systems inside the human body in multiple dimensions. Although the app was not developed for rational drug prescription, it allowed the physicians to reflect on possible usage and express their own ideas on how AR could be used. All interviews were performed in Chinese, audio recorded and transcribed verbatim for analysis. The average interview was 30 min, and the longest interview was 54 min. The semi-structured interview guide can be seen in Appendix.

### Data analysis

We analyzed the interview transcripts to answer RQ1 and RQ2 and thus to understand the physicians’ personal paradigms and their expectations of AR for CPD purposes. We used a hybrid thematic analysis approach [26] that involved inductive and deductive coding. To identify design needs, we developed a deductive coding scheme based on four key components of the MARE framework: 1) learners’ personal paradigms, 2) learning assets, 3) learning activities and 4) the learning environment. The learning environments were: affectively oriented environments that affect healthcare learners’ feelings, perceptually oriented environments used for observations, symbolically oriented environments that are particularly used for thinking and behaviorally oriented environments that are for practicing, or doing [19]. According to the MARE framework, the affectively oriented environments could be well suited to support the learning of new attitudes and could help physicians share values and feelings from their own experiences through different learning activities. The perceptually oriented environments could be useful for examining problem-solving strategies and help physicians change their habit of misusing antibiotics. The symbolically oriented environments could be of value to provide new knowledge to physicians and guide them to develop abilities, and behaviorally oriented environments could be beneficial for physicians to practice their learning and to reflect upon their practices [19]. The coding scheme and its relation to the research questions RQ1 and RQ2 are presented in Table 1.

**Table 1:** Coding scheme based on the MARE design framework [19]

Research questions and definition of key components	Category	Sub-category
RQ1: Personal paradigm: “his or her personal style of diagnosis, treatment, prescription and choice of drugs” (Zhu et al., [19], p. 8).	P-diagnosis	Step 1 Record the patient’s medical history through communicating and inquiring Step 2 Conduct physical examination, including clinical symptoms and signs Step 3 Select laboratory tests and interpret results Step 4 Use diagnostic facilities Step 5 Verify the suitability of your P-diagnosis Step 6 Define the patient’s problem Step 7 Specify the therapeutic objective
	P-treatment	Step 1 Specify treatment objective Step 2 Verify the suitability of your P-treatment Step 3 Start the treatment Step 4 Provide information, instructions and warnings to the patient Step 5 Monitor treatment
	P-drug	Step 1 Specify drug objective Step 2 Make an inventory of groups of drugs that are effective Step 3 Choose an effective group according to the relevant criteria Step 4 Choose a P-drug
	P-prescription	Step 1 Specify prescribing objective Step 2 Choose P-treatment Step 3 Choose P-drug Step 4 Write the prescription Step 5 Provide information, instructions and warnings to the patient Step 6 Monitor the drug treatment effect
RQ2: Expectations of ARAR learning environments: “the conditions and external stimuli that facilitate learning and modify the learners’ paradigms” (Zhu et al., [19], p. 10) AR learning activities: “the approach by which learners obtain meaning from learning material, context, and other people in the learning environment” (Zhu et al., [19], p. 10) AR learning assets: “different media forms, such as text, sound and video that are used to provide the learning content” (Zhu et al., [19], p. 10)	AR learning environments	Affectively oriented Perceptually oriented Symbolically oriented Behaviorally oriented
	AR learning activities	
	AR learning assets	

The qualitative data analysis program NVivo (Version 10; QSR International, Doncaster, VIC, Australia) was used as a management and support tool for transcribing, coding and analyzing the data. The first author (EZ) was immersed in the material by transcribing the interviews and reading the transcripts. Each interview was saved as a separate file in Nvivo. The coding scheme was entered as a node hierarchy by EZ. First, four of the eleven transcripts were coded by tagging meaningful units of text with the predefined nodes. New nodes were created inductively when necessary. The reliability of the coding scheme was discussed with a domain expert and a native Chinese speaker. The results were compared and modified to the predetermined code manual. Thereafter, the remaining transcripts were coded by EZ. The coding results were translated into English and discussed with the co-authors. Uncertainties were resolved in discussion with the co-authors. Then we summarized the data and identified initial themes. The themes were discussed among all authors who added coding through inductive analysis.

Ethical approval for this study was obtained from the Tongji Medical College of Huazhong University of Science & Technology’s (China) ethics committee in November 2012.

## Results

### Basic information about the respondents

The training and background of the respondents is shown in Table 2. Note: In China, dentists are seen as physicians and are allowed to treat non-dental patients in some CHCSs. Additionally, one physician (P7) had studied the National Essential Drug List recently, and another (P9) was participating in ongoing GP in-service training and regularly discussed AMR problems with his supervisor.

**Table 2:** Training and background of the respondents

Interviewee	CHCS	Role in CHCS	Gender	Graduated	Learning Subject^a^
P1	A	Internist	Female	2008	Internal medicine
P2	B	Surgeon	Male	2012	Clinical medicine
P3	A	Dentist	Female	1995	Oral medicine
P4	B	GP	Female	Unassigned	GP
P5	A	Internist	Male	1993	Clinical medicine
P6	C	Chronic disease management	Female	2008	Clinical medicine
P7	A	Surgeon	Female	2004	Clinical medicine
P8	C	Chronic disease management	Female	2007	Clinical medicine
P9	C	GP	Male	1998	Community medicine
P10	A	Pediatrician	Female	1984	Clinical medicine
P11	A	Internist	Female	2008	Clinical medicine

^a^Learning subject was reported by the physicians

### The physicians’ personal paradigms of prescribing antibiotics

Based on the interviews, all categories but not all of the sub-categories in the coding scheme were addressed by the interviewees when they described the therapeutic process with antibiotics (see Table 3). The paradigms for prescribing antibiotics are presented as P-diagnosis, P-treatment, P-prescription and P-drug according to the coding scheme. The step “Specify the objective” was missing in each category, which indicates that the physicians did not set up clear objectives when they diagnosed and treated patients in clinical practice. We also found that the physicians lacked familiarity with the national guidelines for using antibiotics.

**Table 3:** Coding examples of personal paradigm steps and respondents

Category	Sub-category	Respondents per step			Coding example	Possible problem
		Mentioned	Not mentioned	Complained/ neglected/ trivialized		
P-diagnosis	Step 1: Record the patient’s medical history through communicating and inquiring	P1,4–8,10	P2–3,9,11	P1,4–5	But now the situation sometimes is that the patients already have taken pills including antibiotics when they come to our CHCS. We think that the patient doesn’t require antibiotics, but the patient’s family insists we do so.(P1)	Insufficient communication with patient
	Step 2: Conduct physical examination, including clinical symptoms and signs	P1–3,5–11	P4	–		
	Step 3: Select laboratory tests and interpret results	P1–11	–	P1,3–5,7–9	This needs a blood test… We never grow bacterial cultures because our community hospital does not have the equipment to do it. (P3)	Lack of necessary tests
				P5,7,10–11	It (laboratory tests) is not used every time; we diagnose according to our own experience sometimes. (P10)	Negligence of tests
	Step 4: Use diagnostic facilities	P9–11	–	–		
	Step 5: Verify the suitability of your P-diagnosis	P1–2, P4–11	P3	P6	Many primary physicians, both the village physicians and township physicians, lack knowledge of the diseases. For example, they do not even know what disease the patient had after the patient’s recovery. These things have happened. (P6)	Uncertainty about diagnosis
				P1–2, P4–5, P9	Diagnosis is not very important. Diagnosis is easy because the diseases which we treat in our CHCS are mostly respiratory tract infection and acute gastroenteritis. (P4)	Negligence of diagnosis
	Step 6: Define the patient’s problem	P1–11	–	–		
	Step 7: Specify the therapeutic objective	–	P1–11	–		
P-treatment	Step 1: Specify treatment objective	–	P1–11	–		
	Step 2: Verify the suitability of your P-treatment	P1–11	–	P2–3,5–11	We know very little about AMR in our geographic region right now since we cannot monitor AMR. (P5)	Lack of knowledge
				P1, P4	We learn more every time. But even though we learn, the antibiotic is still being used. (P4)	
	*Step 3: Start the treatment	P1–11	–	P1–2,4-6,8–10	Respiratory disease such as bronchitis and upper respiratory tract infections are usually treated with antibiotics. A lot of antibiotics are used in surgery. (P2)	Antibiotic treatment issue
	Step 4: Provide information, instructions and warnings to the patient	P1–2,4-7,11	P3,8–10	P1,4–5,7,11	I told them that ‘you are college students, you should search online,’ but they still not understand. (P4)	Insufficient communication with patient
				P2,4,6	Patients need to have a reasonable level of education about their health through a health class at CHCSs. (P6)	Lack of support/education for patients
	Step 5: Monitor treatment	P1,4–7,10–11	P2–3,8–9	–		
P-drug	Step 1: Specify drug objective	–	P1–11	–		
	Step 2: Make an inventory of groups of drugs that are effective	P1–11	–	P3–4,10	Oral infection is usually treated with broad-spectrum antibiotics and metronidazole. (P3)	Preference for broad-spectrum antibiotics
	Step 3: Choose an effective group according to the relevant criteria	P1–11	–	–		
	Step 4: Choose a P-drug	P1–7,9	8,10–11	P2,4–5,9	We use the recommended drug, and also consider the patient’s financial situation. Both are important factors. It does not matter if they are not sensitive to the cost of the drug. (P9)	Preference for expensive drug
P-prescription	Step 1: Specify prescribing objective	–	P1–11	–		
	*Step 2: Choose P-treatment	P1–11	–	P1–2,4-6,8–11	You cannot wait for the test results before you treat the patient. Bacteria multiply very quickly… I have prescribed some antibiotics infusion for patients who demanded aggressively and made me feel vexed. (P11)	Antibiotic treatment issue
	Step 3: Choose P-drug	P1–11	–	P2,4–5,9,11	It would be better if patients took oral antibiotics early on. The pollution is still too serious in China. (P4)	Abuse of antibiotics
	Step 4: Write the prescription	P4,9,11	P1–3, 5–8, 10	–		
	Step 5: Provide information, instructions and warnings to the patient	–	P1–11	–		
	Step 6: Monitor the drug treatment effect	P1,4–6,8,10–11	P2–3,5,7,9	P11	You should consider stopping to use antibiotics if symptoms disappear, for example pneumonia, if the fever has abated but the patient still has a little cough and we find no rales…You should start the treatment early as well as stop it early. (P11)	Pharmacokinetics

*these two steps might cross coding

#### P-diagnosis

The physicians participating in this study reported that the process for diagnosing the patient and whether the patient needs to be treated with antibiotics was mainly performed in the first three of the seven P-diagnosis steps (see Table 3). At the same time, they experienced that it was difficult to perform Step 3, and there were situations when the physicians used only Step 1 and Step 2. However, there were also physicians who mentioned that they sometimes used methods such as ultrasound, fluoroscopy and X-ray imaging, which correspond to Step 4. Thus, the physicians applied different paths of P-diagnosis to define patients’ problems in Step 6, and the physicians used the patients’ results to choose treatments. When using different paths, some important steps could be missed that might lead to misdiagnosis and the misuse of antibiotics, for example, prescribing antibiotics without knowing the laboratory test results (P-prescription step 2 Coding example P11).

The results revealed several personal paradigm issues in relation to set a proper diagnosis. The first issue identified was related to the physicians’ attitude to diagnosis. Most physicians reported that diagnosing patients was easy and that they had the clinical experiences to do so; only one physician reported that the physicians needed to improve their diagnostic skills. As the majority of the physicians thought diagnosing was easy and less important, they might not verify the suitability of the P-diagnosis (P-diagnosis step 5 Coding example P4).

The second personal paradigm problem was diagnosis uncertainty related to the fact that important laboratory tests often were neglected. They trusted their experience more than the laboratory tests (P-diagnosis step 3 Coding example P10), and one reason mentioned was insufficient laboratory equipment in their clinic.

The interviewees complained about patients taking unnecessary antibiotics for a common cold before coming to the CHCSs, due to leftover pills at home. Three physicians said that they explained to patients that there is no need for antibiotics for such conditions, but that they might prescribe antibiotics anyway if the patients insisted (!).

#### P-treatment

As showed in Table 3, except for Step 1, all the other steps of P-treatment were present in the physicians’ personal paradigms. The physicians reported that the treatment depended on the diagnosis and how they defined the patient’s problem. The outcome could be a referral, no antibiotic therapy or antibiotic treatment as in step 3, and could be changed after monitoring the patient’s recovery. Step 2 of P-treatment varied among the physicians and they realized the problem of antibiotic treatment. Improving their treatment competence and prudent prescribing were regarded as important. Physicians experienced problems when communicating with patients in Step 4. Physicians suggested that patients needed to be educated that antibiotic therapy is not always indicated.

The physicians mentioned frequently that common diseases they often treated with antibiotics were upper respiratory tract infections, bronchitis, pneumonia, acute gastroenteritis, appendicitis, soft tissue infection, urinary tract infections and oral infections. However, not all such conditions need antibiotics treatment. For example, one physician reported that most of the upper respiratory tract infections are caused by viruses, and another mentioned that acute gastroenteritis could not be treated with antibiotics unless there were signs of bacteria in the stool. Some diseases, for example children with measles, chicken pox and fever, women with menstrual problems or patients with fractures were also treated with antibiotics. Each of above diseases was reported by no more than two physicians. Five physicians mentioned that a common cold could not be treated with antibiotics, but that they could be used if the patient later had a secondary infection.

All physicians said that they realized the problem of misuse and overuse of antibiotics, and that they should use antibiotics more carefully. According to the suggestions from some physicians, CPD could be a way to verify the suitability of P-treatment. Two physicians reported that they were gradually changing their minds after training. They reported the importance of improving their competence in using antibiotics, local AMR and other new treatments (P-treatment step 2 Coding example P5). However, physicians felt it was difficult to change their prescription paradigm although they knew that antibiotics were not necessary in many cases (P-treatment step 2 Coding example P4). They also felt pressure from patients and patients’ families to prescribe antibiotics.

Physicians reported the need to educate patients especially about conditions that do not require antibiotic therapy (P-treatment step 4 Coding example P6). They said that they tried to explain patiently to patients. However, they felt that it was hard to communicate with patients who did not understand and did not trust physicians at CHCSs. Thus, the physicians suggested that the public health departments of the CHCSs should educate patients or patients should learn online by themselves (P-treatment step 4 Coding example P4).

#### P-prescription and P-drug

Except for Step 5- Provide information, instructions and warnings to patients, as well as Step 1- Specify prescribing (or drug) objective, we found that the physicians used the other four steps of P-prescription and three steps of P-drug in Table 3. To highlight the clinical relevance of this study, we mixed the P-drug category with the P-prescription category because antibiotics are prescribed by physicians and P-prescription includes choosing a P-drug. Additionally, they mentioned that prescriptions were also checked by the government in order to monitor the use of antibiotics.

Antibiotics were prescribed based on three pathways: if physicians thought, diagnosed or monitored patients were infected with bacteria, if the physicians thought preventing further infection for the patient was necessary or if the patients requested antibiotic treatment (particularly when it came to intravenous infusions and/or they also wanted to avoid conflict with the patient). The last path is obviously characterized by unnecessary prescribing of antibiotics (P-prescription step 2 Coding example P11); the other two paths could also lead to misuse of antibiotics as discussed for P-treatment. For example, one surgeon prescribed antibiotics he believed could prevent infection caused by minor surgery, and three physicians even referred to pollution as a possible cause (P-prescription step 3 Coding example P4).

The physicians often prescribed antibiotics immediately if the physicians diagnosed an infection, and even without waiting for laboratory test results. Moreover, there were not enough different types of antibiotics for physicians to choose among in the CHCSs according to the physicians. Therefore, they often used broad-spectrum antibiotics. Some physicians reported that they sometimes used a combination of two antibiotics and that they would like to learn more about this kind of treatment.

Interestingly, four physicians assumed expensive antibiotics were better than cheap ones in the same class and suggested patients use the expensive one if the patients’ financial situations allowed. (P-drug step 4 Coding example P9). Six physicians believed that patients were treated with more advanced antibiotics in other hospitals.

### Physicians interpreting the national antibiotics guidelines

Surprisingly, none of the physicians in this study was familiar with the official national antibiotics guidelines. One physician stated that she had a guideline book when she was a graduate student several years before but could not remember the name of the book. Several physicians reported that they knew that guidelines for using antibiotics were available online but were unable to say where or what guidelines they had seen. Three physicians mentioned that there was no guideline for them; however, they used the Pharmacopoeia for prescribing. Five physicians pointed out the guidelines for other things such as the National Essential Drug List, a handbook for inpatients, the sign of blood test etc. Moreover, some physicians did not even trust guidelines:

“No, there are no guidelines. We all prescribe based on our experience, and I do not believe there are any guidelines. Guidelines - those things are used to coax people.” (P9)

### The physicians’ personal paradigm problems and learning objectives

Table 4 provides an overview of the learning needs we identified for the physicians in this study. Guided by the MARE framework, we identified ten personal paradigm problems (PPPs) and 13 important learning objectives (LOs) for the physicians. We identified the “problems” from our empirical material when the respondents did not follow the WHO good prescribing standard. Unfamiliarity with guidelines that is one personal paradigm problem (PPP3) affected not only the physicians’ P-diagnosis but also their P-treatment. Two learning objectives (LO3 and LO4) referred to the importance of adhering to established guidelines. With this summary of the interview results, we have taken a step toward understanding physicians’ CPD design needs.

**Table 4:** Personal paradigm problems related to learning objectives and abilities, based on the MARE design framework [19]

Personal paradigm category	Respondents	Personal paradigm problem (PPP)	Learning objective (LO)	Type of expected ability
P-diagnosis	P 1,3-5,7-9,10–11	1. Neglected to conduct the necessary laboratory tests	1. Implementing microbiological and other investigations to diagnose	Knowledge: Skill
	P1–2,4-5,9	2. paid no attention to the importance of diagnosis	2. Maintaining patient respect in line with best practice, regulatory standards, and contractual requirements	Action: Attitude
	P1–11	3. Unversed in the official antibiotics guideline document	3. Stating national public health antibiotics guidelines	Knowledge: Cognition
			4. Selecting and prescribing antibiotic therapy according to national/local practice guidelines	Competence: Cognition
P-treatment	P1–2,4-6,8–10	4. Prescribed antibiotics for no obvious evidence of bacterial infection	5. Not initiating antibiotic treatment in the absence of bacterial infection	Competence: Attitude
	P1,4–5,7,11	5. Failed to communicate with patients about no antibiotic treatments	6. Mastering delayed antibiotic therapy and negotiation with the patient	Performance: Skill
			7. Educating patients and their caregivers, nurses and other support clinical staff	Action: Cognition
P-prescription and P-drug	P4–6,8–11	6. Did not know local AMR patterns	8. Using local microbial−/antimicrobial-susceptibility patterns when conducting empirical treatments	Competence: Cognition
	P2,4–5,9,11	7. Prescribed antibiotics without waiting for laboratory test results	9. Understanding the importance of taking microbiological samples for culture before starting antibiotic therapy	Knowledge: Attitude
	P3–4,10	8. Preferred to prescribe broad-spectrum antibiotics	10. Avoiding the unnecessary use of broad-spectrum antibiotics	Competence: Attitude
	P2,4–5,9	9. Preferred to prescribe expensive antibiotics	11. Working within the ethical code of conduct	Performance: Attitude
			12. Applying legal and ethical frameworks affecting prescribing practice	Performance: Attitude
	P11	10. Stopped antibiotic treatment when the symptoms disappeared	13. Constructing the prescription for an antimicrobial with its pharmacokinetics and knowing how this affects the choice of dosage regimen	Competence: Cognition

Table 5 shows each physician’s personal paradigm problems. Most of the physicians seemed to have different issues in their prescribing practice. Only one surgeon (P7) that had learned the national list of basic drugs did not report antibiotics overuse except pushed by patients (PPP5).

**Table 5:** The background of the respondents and their potential personal paradigm problems (PPPs)

Participants	PPP1	PPP2	PPP3	PPP4	PPP5	PPP6	PPP7	PPP8	PPP9	PPP10
P1 Internist										
Internal medicine	X	X	X	X	X					
P2 Surgeon										
Clinical medicine		X	X	X			X		X	
P3 Dentist										
Oral medicine	X		X					X		
P4 GP										
GP	X	X	X	X	X	X	X	X	X	
P5 Internist										
Clinical medicine	X	X	X	X	X	X	X		X	
P6 CDM										
Clinical medicine			X	X		X				
P7 Surgeon										
Clinical medicine	X		X		X					
P8 CDM										
Clinical medicine	X		X	X		X				
P9 GP										
Community medicine	X	X	X	X		X	X		X	
P10 Pediatrician										
Clinical medicine	X		X	X		X		X		
P11 Internist										
Clinical medicine	X		X		X	X	X			X

*CDM* Chronic disease management

### Physicians’ expectations of the functionality and content of AR applications

In the second part of the study the physicians were asked to reflect upon what functionality and content they would like a future AR-based CPD to have. As expected, different physicians had different expectations for AR applications based on learning assets, learning environments and learning activities. The results are illustrated in Table 6 below.

**Table 6:** Coding examples of expectations of AR and respondents

Category	Sub-category	Respondents^a^			Coding example
		Not mentioned	Suggestions		
AR learning environments	Affectively oriented	P2–3,5-6,8	P1,7,9–11	Positive	It would be more impressive if you show the whole process of design making to physicians. It needs to be developed and studied by people such as you. (P11)
	Perceptually oriented	P3,6–7,9–10	P1–2, 5, 8,11	Positive	It may also be necessary for some animation to demonstrate the surgical process, inflammatory response, and immune response in treatment of the bacteria with antibiotic.(P2)
	Symbolically oriented	P7, P10–11	P1–3,8	Positive	We understand better through reading a text. (P8)
			P5–6,9	Negative	It is boring to read text, and we do not see the anatomy clearly which makes it hard to remember. (P5)
	Behaviorally oriented	P2,5–9,11	P10	Positive	All are ok; the key is that you need time to develop. Then we can simulate. (P10)
			P1,3	Negative	I think that it is certainly not possible for us to use it for practical hands-on skill. (P3)
AR learning activities		P3–4,8	P1–2,5-7,9–11		It would be better if it had a certain search function similar to in English-Chinese dictionary…I can search classification of antibiotics and diseases…For example, If I know it is a *Staphylococcus aureus* infection, I can search for these bacteria, and which drugs that can effectively kill the bacteria and if there are corresponding tests etc. (P2)
AR learning assets		P4,10	P1–3,9	Text for new antibiotics, guideline	Now we certainly need some training on new drugs and for what indications it should be used and what the contraindications are. This is a weakness because of our CHCS… I like text. (P3)
			P2,5,7–9	3D for AMR and infection	3D is certainly better, especially if you use dynamic 3D AR to show the mechanism of how antibiotics work….(P7)
			P1–2,6,11	Multimedia for competence	For learning, we need more training on diagnostics ability including improving our comprehensive knowledge … I think that video, pictures and text can be combined together to enhance learning. (P6).

^a^P4 reported she did not care for any kind of learning environment and had no idea for the learning activities

#### AR learning assets

The physicians’ expectations for a possible AR-based learning asset varied because they preferred different types of media. After the physicians had been introduced to the APP PlayAR Human Anatomy 4D, they reported that multidimensional AR technology could be used in different ways for them to better understand AMR, help them communicate with patients and show the site of infection and treatment with antibiotics, for example, a simulation of the AMR mechanism (AR learning assets Coding example P7). Moreover, the physicians reported that various media should be combined in an AR application to improve their competence (AR learning assets Coding example P6), for example, showing the patient’s hoarse voice and picture of a sore throat together with the real person’s body. However, one physician reported that instead of an AR app she would prefer simple text-based information describing new antibiotics, indications and contraindications (AR learning assets Coding example P3) and one physician would like to use AR and simple text.

#### AR learning environments and learning activities

Most physicians preferred different types of learning environments. Some physicians stated that it did not matter what kind of learning environment they used. Only a few mentioned the need of a behaviorally oriented leaning environment; only one physician objected to e-learning environments for training on how to use antibiotics and preferred traditional learning materials instead. However, physicians mentioned affectively oriented and perceptually oriented environments. Furthermore, some physicians argued for a symbolically oriented environment although others believed such an environment was boring. Different learning activities were expected in the different learning environments, mainly based on the physicians’ current learning experience with technology.

Most of the physicians expected the environment to be attractive and interesting to prevent them from getting bored. It was also assumed that it should be impressive (Affectively oriented Coding example P11). A couple of the physicians considered also humor to be a good environmental element. Storytelling, collaborating and sharing with peers were suggested as learning activities. For example, the physicians had the experience of sharing work stories and knowledge through social media.

The physicians thought that simulations should show how antibiotics and the treatment process work for different health conditions (Perceptually oriented Coding example P2). They suggested that demonstrations and observation, including case studies, were good learning activities in this perceptually oriented environment.

There were different options for the symbolically oriented environment according to the physicians in this study. Searching online and surfing was a common activity for physicians who had to face uncertain situations. Although it was a custom to read, four of them regarded reading as boring if only the symbolically oriented environment was used (Symbolically oriented Coding example P5).

Few physicians mentioned the need for a behaviorally oriented environment, and the doubtful thought was that it is impossible to teach the use of antibiotics in a behaviorally oriented environment (Behaviorally oriented Coding example P3).

## Discussion

AMR is estimated to lead to 10 million deaths every year and cost up to USD 100 trillion by 2050 if the threat continues [27]. Overuse of antimicrobials and inappropriate prescribing lead to increasing AMR [28–30]. Physicians’ decisions to prescribe antibiotics are affected not only by intrinsic factors, including clinical practice, education, attitude and uncertain diagnosis, but also by extrinsic factors related to demands from and expectations of patients and the healthcare system [18]. Through the application of the MARE framework, we were able to identify primary care physicians’ prescription issues and need for educational interventions. Moreover, we tried to understand how AR could be used to support their learning needs. Different physicians had different expectations for a possible AR-based education intervention, with respect to content, learning assets, learning environment and learning activity.

### Comparison with other studies

To our knowledge, this is the first study to explore the need for education and ways that AR might be used with respect to physicians’ expectations and their personal paradigms of prescribing antibiotics. Several studies have explored physicians’ antibiotic prescription and effective interventions, including education. However, those studies did not have the same focus as this study. In this study, we showed how to use the design framework MARE [19] to identify physicians’ paradigm problems and learning needs in connection with their decision-making process for antibiotic treatment of patients, as well as design considerations for AR-based education aiming to improve the decision-making process. The physicians suggested some learning activities based on their learning experiences and the technology tools they currently used. Their limited suggestions for learning activities in AR-based CPD showed that learner-centered design also needs educational experts for the design [31]. Many possible learning activities that could be used in different learning environments are included in the MARE framework. Moreover, the physicians shared meaningful expectations regarding learning assets we had not detailed in the framework.

We used a qualitative study to describe primary care physicians’ personal paradigms in the decision making process. A similar study of GPs’ decision making regarding prescribing antibiotics was conducted in Iceland more than 10 years ago [32]. We agree with their finding that physicians often use three main paths to prescribe antibiotics: (1) when GPs know or think that the patient was infected by bacteria, (2) when GPs lack time or are too tired to explain the unnecessary antibiotic treatment for the viral infection to patients and (3) when GPs respect patient autonomy too much. However, we suggest that the latter two paths related to patients could be combined into one because both address the communication problem. Moreover, we found another path, which is prescription to prevent infection. We also identified 13 learning objectives based on the physicians’ personal prescription problems. The physicians in this study had more than 5 years’ clinical experience except one who graduated in 2012. Thus, we considered that the physicians would have the basic abilities described within the MARE design framework, if they were not obviously wrong in their reporting according to the framework. A systematic review reported the overuse of antibiotics across different levels of hospitals and in primary care in China [17]. In addition, a qualitative study in China showed that incorrect knowledge and misconceptions about antibiotic use were widely disseminated and shared by physicians [33]. The gap between physicians’ knowledge and their actual antibiotic treatment of common colds in China was shown in a quantitative study [34].

Other qualitative studies focused on analyzing the factors of unnecessary prescribing or overprescribing of antibiotics by physicians [35]. Physicians’ intrinsic factors were summarized by two systematic reviews as physicians’ uncertain diagnosis and attitude, including ignorance, fear, confidence, complacency, indifference and responsibility for others [18, 36]. These factors were also shown in our interviewees’ decision-making process although they thought diagnosing patients were easy.

The physicians in this study experienced difficulties with patients who insisted on antibiotics. The patients’ requirements, which have been shown in many other studies, were mainly mentioned as an extrinsic factor in this study. Improving patients’ knowledge of appropriate antibiotics was expected by physicians and was also used in an audit study in China [37]. They found that physicians’ antibiotic prescription rates were lowered and patients’ drug expenditures decreased when patients received appropriate knowledge of antibiotics. Educational interventions for primary care physicians and patients have reported positive results to improve antibiotic use [38].

AR has been used in medical education for some years [14]. Several studies have been conducted to evaluate AR - user acceptance, usability or effectiveness - by medical students or health care professionals after the AR applications were developed [39–41]. However, we did not find other studies investigating the AR design needs from the learners’ view in the early design stage. We also did not find AR being used in primary care for improving antibiotics use.

### Implications for design

**Fig. 1:**
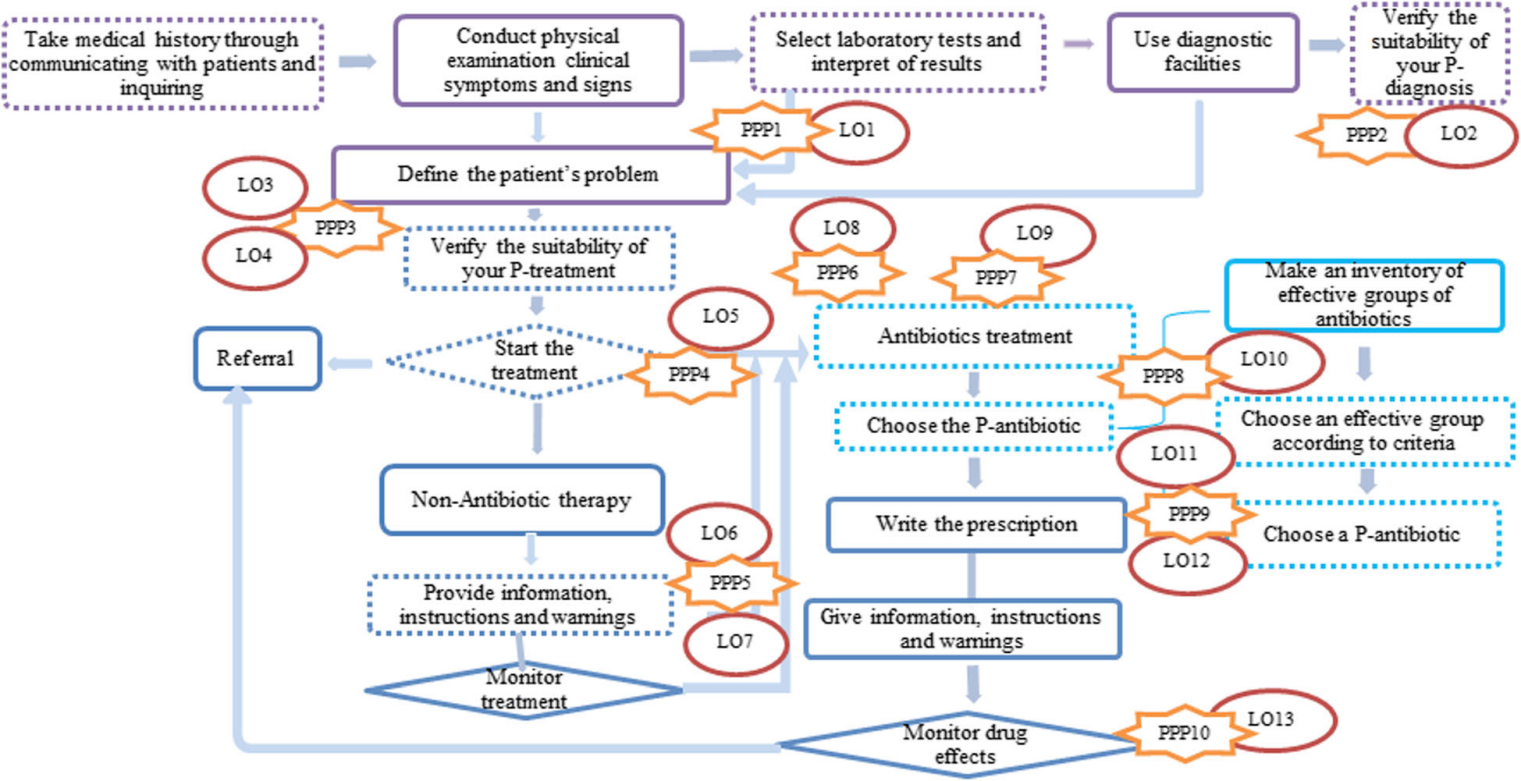
Application of the MARE framework on the aggregated personal paradigms

Based on our results of learning objectives and physicians’ expectations, AR might provide a potential educational tool for primary care physicians to modify their personal paradigms concerning prescription of antibiotics. We summarize the personal paradigms issues and related learning objectives in the decision making process in Fig. 1. In the figure, the physicians’ aggregated personal paradigms are illustrated, and the identified personal paradigm problems (PPPs) and learning objectives (LOs) are indicated. The dotted lines in the figure mean that physicians have complained, neglected or trivialized those steps. The explosion symbols with PPPs show the possible problems, and the related LOs use oval symbols to illustrate the learning needs.

The physicians expected AR to help them gain a better understanding about AMR and infections, which corresponds to LO3–4 and LO8. Some other studies have discovered the AMR patterns in China as well as in Wuhan [42, 43]. This kind of data could be used to visualize the AMR patterns on a Chinese map and a city map, for the physicians to compare their local AMR patterns with the ones for the whole country. Similarly, we could also illustrate the guidelines as a cognition map. For example, clinic practice guidelines for diagnosis and treatment of acute bronchitis, which have been published by the professional associations from China as well as other countries [44–47], could be used to illustrate the cognition map within the WHO guide for rational process. Through the use of AR, objects and symbols could be added to a text description of the rational process, with special attention directed to certain steps, including labels and close-up views with additional information required to perform the work, showing how to practically proceed with examinations and lab tests, for example.

Another learning need that could be supported by AR concerns the physicians’ ability to communicate with the patients, together with the patients’ cognitive view of treatments. AR applications could be used as a tool to help the physicians explain treatments to patients, and thereby improve the decision-making. This would fit the LO6 and LO7 related to PPP5 that concerned five of the participants. When the physicians need to educate patients, for example explaining no antibiotic treatment for bronchitis, they could use a practical demonstration technique by asking the patient to use a smartphone to track a person’s chest part. A 3D animation of bronchi would then be combined with the person’s chest part on the smartphone. The patient could use the animation to learn about the causes of acute bronchitis and also click the buttons of antibiotics and alternative treatments to see the expected results.

We can design these kinds of AR environments as the physicians most often suggested affectively oriented and perceptually oriented environments, and they accepted the symbolically oriented environments if combined with others. Among the learning activities the physicians suggested also storytelling, collaborating and sharing with peers. Such activities are important when trying to manage community attitudes and behavior concerning the rational therapeutic process (LO2, LO5 and LO9–12, for example). Other types of e-learning, such as tools for web-based communication and collaboration, are therefore suggested to be used together with AR.

How to design learning environments for technology enhanced learning is considered an important aspect in design science of education [48]. Other types of e-learning could also provide different learning environments, for example, computer-based learning (CBL) which could be either online or offline. Cook [49] suggested that CBL should be adapted in response to individual differences, such as previous knowledge computer experience, or learning style. He also suggested researching how CBL could be integrated with physicians’ everyday clinical practices and their clinical institutions to facilitate learning. This study provided understanding of physicians’ learning needs and possible AR learning environments from the physicians’ point of view. Moreover, combining AR with traditional CBL could provide more integrated learning and clinical environments, which is in line with what Cook suggested.

In order to design an effective CPD using e-learning and AR technology to improve GPs’ rational use of antibiotics, we suggest that the MARE framework [19] be applied. In this article, we have shown that the studied physicians were not only unfamiliar with national guidelines and local AMR patterns, but also that there were problems related to their decision making for P-diagnosis, P-treatment, P-prescription and P-drug. Moreover, the physicians also provided meaningful expectations and ideas for e-learning and AR-based education. Although some physicians claimed that they needed to learn only about new antibiotics or guidelines, we found that their problems were more systematic. As physicians had different personal paradigms and learning preferences, the design should be flexible to the scaffold.

### Methodological considerations

This study was conducted in three CHCSs in Wuhan, China. Rigor for a qualitative study should be ensured through addressing the trustworthiness of credibility, transferability, dependability and confirmability [50]. Some strategies to ensure the trustworthiness suggested by Shenton were used in this study [51]. The first author was living in Wuhan at the time of the study, and she had known some of the physicians for many years. Other participants were introduced by these physicians. This familiarity can help ensure honesty from the interviewees and ensure the credibility of the study. She also used interactive questioning and reflective commentary to improve credibility during the data collection.

The first author’s continual immersion in the data and her discussion with co-authors during the analysis also ensured credibility. In the beginning of the analysis, a discussion with a Chinese domain expert who has a PhD in clinical pharmacology and is an associate professor at Karolinska Institutet (KI) was performed to further improve the credibility. The three CHCSs were located in different parts of Wuhan, which provides site triangulation. This triangulation is good for credibility, as well as confirmability. Using the software NVivo provided a good audit trail to discuss with a domain expert for the confirmability. The first author also translated the coding summary as an audit trail to check by co-authors and the domain expert. Dependability was maintained through the description of our research design, data collection and the result implications. The physicians’ personal paradigm of using antibiotics and their expectations for AR-based CPD which we analyzed in the CHCSs in Wuhan might not be applicable to other contexts. However, common problems, which we found in this study and were confirmed by other studies, showed that the MARE framework could be applied in other situations. The background data and the details described in this study can be used for comparison with other studies for the transferability.

### Limitations

This study provides an understanding of physicians’ personal paradigms of prescribing antibiotics in Chinese urban primary care settings and the physicians’ expectations for AR-based education applications. However, the study has several limitations. First, this study mainly focused on interviews with physicians in primary care, meaning that it might not be applicable in other settings. It could also be interesting to interview patients to receive their view. Second, in this qualitative study, a limited number of primary care physicians were interviewed, and we did not include primary care physicians in for example private clinics who might have another paradigm for prescribing antibiotics and issues. Third, we mainly focused on AR in this study, and traditional forms of CBL were only briefly discussed in connection with AR. Last, we used convenience sampling and snowball sampling as well as a small sampling, which might have influenced the results [24].

## Conclusions

In this study, we have interviewed primary care physicians in China to discover their personal paradigms for prescribing antibiotics and their expectations for a possible learning intervention based on AR. The concept of personal paradigms can be used to describe the physicians’ decision-making process for diagnosis, treatment and prescription, including the choice of drug, and reveal the problems related to it. Problems identified in the process were uncertain diagnosis, ignorance of national guidelines and the local AMR pattern, reliance on their own experience rather than adherence to guidelines, complacency in their diagnosis and failure to communicate with patients about non-antibiotic treatments. With the application of the theoretical framework for MARE, the physicians’ personal paradigm problems, learning needs and expectations of continuous AR-based education with respect to contents, learning assets, learning environments and learning activities were found. The results revealed considerable learning needs to improve the decision-making process and avoid misuse of antibiotics in the treatment of primary care patients in China. As a possible next step, we could use the results to design prototypes, to help physicians develop the expected abilities and modify their personal paradigms. Since their expectations and their personal paradigm problems could be different and they were not very familiar with advanced technology use, the design should be flexible and creative. The prototypes could use the application of the MARE framework that was elaborated on in this article as a scaffold for the design of CPD interventions.

## Appendix

**Table 7:** The guide questions for interview (translated from Chinese)

1) When did you graduate, and what was your main subject in medical school?	
2) For what types of diseases do you prescribe antibiotics in the CHCS?	
3) How do you diagnose these diseases?	
4) How do you decide if patients need antibiotic treatment?	
5) What kinds of antibiotics do you use to treat these diseases?	
6) How do you select antibiotics, and what kind of knowledge do you need to master the rational use of antibiotics?	
7) Which processes do you feel are important for your rational use of antibiotics during diagnosis, treatment, and choosing and prescribing antibiotics? What competence do you need for it?	
8) What aspects do you feel confident in, and what aspects do you feel you need additional learning to prescribe antibiotics?	
9) What guidelines do you use when prescribing antibiotics?	
10) What kind of learning method would you prefer if we developed an AR application for use on your smart phone?	
11) There are many ways and different media to show the learning content. Which ones are your favorites?	

## Abbreviations

3D: Three-dimensional
AMR: Antimicrobial resistance
AR: Augmented reality
CBL: Computer-based learning
CHCS: Community health service centers or stations
CPD: Continuing professional development
GP: General practitioner
LO: Learning objective
MARE: Mobile augmented reality education
PPP: Personal paradigm problem
RQ: Research question
WHO: World Health Organization
